# Valsalva maneuver using a Handmade Device in Supraventricular Tachycardia Reversion; a Quasi Experimental Study

**DOI:** 10.22037/emergency.v5i1.18805

**Published:** 2017-10-15

**Authors:** Maryam Motamedi, Mohammad Ali Akbarzadeh, Saeed Safari, Mehrnoosh Shahhoseini

**Affiliations:** 1 Emergency Department, Shohadaye Tajrish Hospital, Shahid Beheshti University of Medical Sciences, Tehran, Iran.; 2 Cardiovascular Research Center, Modarres Hospital, Shahid Beheshti University of Medical Sciences, Tehran, Iran.

**Keywords:** Vagus nerve stimulation, Valsalva maneuver, tachycardia, supraventricularm arrhythmias, cardiac, emergency service, hospital

## Abstract

**Introduction:**

The use of vagal nerve stimulation is identified as a proper treatment option in patients with stable supraventricular tachycardia (SVT). This study aimed to assess the success of Valsalva maneuver via a handmade device in reversion of SVT.

**Methods:**

In this quasi experimental study, using a handmade device, vagus nerve stimulation was performed for SVT patients presenting to emergency department or cardiac intervention unit and the success rate and its related factors were assessed.

**Results:**

100 patients with the mean age of 53.05 ± 13.70 years were studied (67% female). 12 (12%) cases were unable to do the maneuver. Out of the 88 (88.0%) patients who could perform the maneuver, 75 (85.2%) cases were unsuccessful. Dysrhythmia was controlled in 6 (6.8%) cases on the first attempt and in 7 (8.0%) cases on the second one (14.8% total success rate). 12 of the 13 cases (92.3%) with successful maneuver had history of SVT (p = 0.031). There was not any significant association between success rate and sex (p = 0.084), age (p = 0.744), or other medical histories (p ≥ 0.05).

**Conclusion:**

Based on the results of the present study, the success rate of Valsalva maneuver with the mentioned handmade device was calculated to be 14.8%. The only independent related factor of successful reversion was SVT history.

## Introduction

Supraventricular tachycardia (SVT) comprises a common group of cardiac arrhythmia in patients referring to emergency departments. The overall incidence of this acute phenomenon has been estimated to be 35 cases per 100,000 patients (1). SVT symptoms are often non-specific, which include chest pain, shortness of breath, sense of palpitation, pounding in the neck, and even some degree of psychological disturbances such as anxiety (2). Impulse formation and impulse conduction processes may both be disturbed (3). In fact, three mechanisms of triggered activity, reentry, or increased automaticity with atrioventricular node source or above cardiac tissue source may be involved in SVT (4). Hence, the initial management of SVT involves slow atrioventricular node conduction by evolving pharmacological approaches, mechanical maneuvers, or invasive approaches if required (5-14). 

The use of vagal maneuvers is now identified as a proper treatment option for patients with stable SVT but its success rate varies between 6% and 54% (15-19). There are different options for performing vagal maneuvers including the Valsalva maneuver and carotid sinus massage (20). However, there is no measurable and controlled method in this regard. The present study aimed to assess the success of Valsalva maneuver via a handmade device in reversion of SVT.

## Methods

***Study design and setting***

This quasi experimental multicenter study was performed from Oct 2014 to Oct 2016 at four general educational hospitals (Imam Hossein, Shohadaye Tajrish, Loghman Hakim, and Modarres Hospitals), Tehran, Iran. Using a handmade device, vagus stimulation was performed for patients presenting to emergency department or cardiac intervention unit following SVT. The study protocol was approved by ethics committee of Shahid Beheshti University of Medical Sciences. All researchers adhered to principles of Helsinki declaration and confidentiality of patients’ information.

Participants

The study was conducted on patients >18 years old who consecutively presented to emergency departments of the mentioned hospitals with the initial evidence of SVT (n = 45) or were scheduled for inducing arrhythmia by a cardiologist at cardiac intervention unit (n = 55). 

Patients who were not able to perform the maneuver or did not give consent, those with hemodynamic instability, atrial fibrillation or flutter, malignant hypertension, or other contraindications for performing Valsalva maneuver (such as cerebral AVM, increased intraocular pressure, glaucoma, or third trimester of pregnancy) were excluded. 

***Procedure***

**Figure 1:**
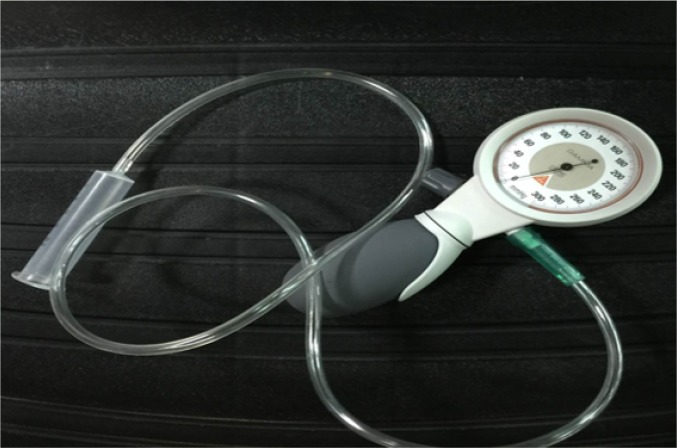
The handmade device used in the present study.

For creating the Valsalva maneuver’s handmade device, the cuff of the sphygmomanometer was disconnected and the device’s pressure monitor was connected to a 90cm oxygen connector, and the end of connector was connected to a 10cc syringe (figure 1).

Maneuver was performed in supine position and with 30 to 50 mmHg pressure for 15 seconds. If the first attempt was unsuccessful, another attempt was made after five minutes. In cases of unsuccessful reversion with two Valsalva maneuvers, pharmacological approach was begun. 

During the maneuver performance, all required supportive instruments including intubation and defibrillation sets were available and the patients were under continuous cardiac and blood pressure monitoring. 

***Outcome***

Achieving sinus rhythm following the first or second maneuver attempt was considered as the main outcome of study (successful maneuver). 

***Statistics analysis***

Results were presented as mean ± standard deviation (SD) or frequencies and percentages. Chi-square or Fisher's exact tests and t test were used for comparisons. Data were analyzed using SPSS 21 statistical software. P values of 0.05 or less were considered statistically significant. 

## Results

***Baseline characteristics***

100 patients with the mean age of 53.05 ± 13.70 years (22 - 85) were studied (67% female). The baseline characteristics of the subjects are summarized in Table 1. Most patients were in the age range of 40 – 60 years and 67% of them had history of SVT.

SVT had resulted from atrio-ventricular nodal re-entrant tachycardia (AVNRT) in 59%, atrio-ventricular reentrant tachycardia (AVRT) in 14%, and unknown origin in 27%. 

***Maneuver Outcome***

12 (12%) cases were unable to do the maneuver. Out of the 88 (88.0%) patients who could perform the maneuver, 75 (85.2%) cases were unsuccessful. Dysrhythmia was controlled in 6 (6.8%) cases at the first attempt and in 7 (8.0%) cases at the second one (14.8% total success rate). 

12 of the 13 cases (92.3%) of successful maneuver had history of SVT (p = 0.031). There was not any significant association between success rate and sex (p = 0.084), age (p = 0.744), or other medical histories (p ≥ 0.05). 

Unable and unsuccessful cases were treated with ablation (52.9%), adenosine (40.4%), amiodarone (4.5%), and verapamil (2.2%). 

## Discussion

Based on the results of the present study, the success rate of Valsalva maneuver with the introduced handmade device was estimated to be 14.8%. The only independent related factor of successful reversion was SVT history.

**Table 1:** Baseline characteristics of the studied patients

**Variables**	**Values**
**Sex**	
Female	67 (67)
Male	33 (33)
**Age (year)**	
20 – 40	16 (16)
40 – 60	50 (50)
≥ 60	34 (34)
**Medical history**	
Hypertension	17 (17)
Diabetes mellitus	19 (19)
Smoking	10 (10)
Ischemic heart disease	17 (17)
SVT	67 (67)
Arrhythmias	6 (6)
Calcium blocker use	11 (11)
Beta blocker use	45 (45)
**Weight (kg)**	73.8 ± 13.8
**Height (cm)**	165.0 ± 8.2
**Heart rate (beats/minute)**	173.3 ± 25.7

*Data were presented as mean ± standard deviation or number (%). *

*SVT: Supraventricular tachycardia.*

Along with medications and ablation, the use of vagal maneuvers has been introduced as an alternative modality leading to reversion of AVRT and AVNRT in up to 54% of affected patients (17, 21). 

Vagal maneuver can be the most successful method to reverse arrhythmias if the maneuver is performed in supine position with adequate intra-thoracic pressure (at least 30 mmHg) for at least 15 seconds (22, 23). Despite considering all the mentioned conditions, the success rate of this maneuver has been considerably low in almost all studies. As shown in our study, the vagal maneuver was successful only in 14.8% of subjects. This rate was about 19.4% in the report by Lim et al. (17). However, in animal and laboratory-based studies as well as in the new modified version of Valsalva maneuver, the rate of reversion was shown to be in the range of 45.9% to 54.3% (1, 21). 

Taylor et al. showed that only few number of physicians give enough instructions to their patients regarding the position, duration, pressure and other characteristics of maneuver (24). 

According to the second finding of our study, with respect to main determinants of the success of Valsalva maneuver to reverse SVT, none of the baseline characteristics, except for previous experiment of SVT, could predict high success rate for this maneuver. 

Higher success of this maneuver to terminate SVT in those with previous SVT might be due to more careful and cautious performance of the maneuver by the specialists in this high risk group or more excitability of cardiac conductive system of those cases by inducing maneuver. 

It seems that, a majority of patients who suffer from SVT may not benefit from Valsalva maneuver and thus, employing the modified maneuver is more recommended. Higher success of this maneuver in patients with history of SVT requires more evaluation. Therefore, further studies with larger and heterogonous samples and proper follow up are recommended.

***Limitation***

In this study, more than half of the participants were patients with induced arrhythmia by cardiologist, which may affect the response to treatment. 

## Conclusion

Based on the results of the present study, the success rate of Valsalva maneuver with the mentioned handmade device was calculated to be 14.8%. The only independent related factor of successful reversion was SVT history.
